# FGD1 restrains actomyosin contractility to promote suprabasal junction maturation in stratified keratinocytes

**DOI:** 10.1242/jcs.264937

**Published:** 2026-09-23

**Authors:** Diana I. Hotea, Alexandra Jetté, Amy Beaudin, Maité Lauzon, Krithika Kalyanakrishnan, Mélanie Laurin

**Affiliations:** ^1^ Département de biologie moléculaire, biochimie médicale et pathologie, Faculté de médecine, Université Laval, Québec, Québec G1V 0A6, Canada; ^2^ Oncology Research Axis, CHU de Québec - Université Laval Research Center, Québec, Québec G1V 0A6, Canada; ^3^ Centre de recherche sur le cancer de l'Université Laval (CRC), Québec, Québec G1V 0A6, Canada; ^4^ Centre de recherche en organogénèse expérimentale (LOEX), Québec, Québec G1V 0A6, Canada

**Keywords:** FGD1, CDC42 signaling, Actomyosin contractility, Adherens junctions, Keratinocyte, Junction maturation

## Abstract

The epidermis is a stratified epithelium in which keratinocytes differentiate as they move from basal to suprabasal layers, a process during which tissue integrity relies on the coordinated remodeling of intercellular junctions and the cytoskeleton. Unlike simple epithelia, which form a continuous zonula adherens, suprabasal cells in the epidermis lack a canonical junction belt, with E-cadherin-based junctions distributed across the entire cell surface, highlighting distinct regulatory mechanisms that remain poorly understood. Here, we identify the CDC42 guanine nucleotide exchange factor FGD1, mutated in Aarskog–Scott syndrome, as a regulator of suprabasal junction architecture. Using stratified human keratinocytes and quantitative imaging, we show that loss of FGD1 induces elongated radial actin bundles and tortuous, tension-associated adherens junctions resembling the stress fiber-anchored discontinuous (focal) adherens junctions described in endothelial cells. These defects are accompanied by altered tissue mechanics. Inhibition of ROCK or myosin II restores junction organization, demonstrating that FGD1 restrains actomyosin contractility to promote suprabasal junction maturation. Several disease-associated FGD1 variants fail to rescue these defects. Together, our findings identify FGD1 as a regulator of suprabasal junction maturation.

## Teaser

**Summary:** We identify FGD1 as a regulator of suprabasal adherens junction remodeling that restrains actomyosin contractility to coordinate cytoskeletal organization and epithelial tissue mechanics.

## INTRODUCTION

The epidermis is the primary barrier of the body against environmental insults and consists of a stratified squamous epithelium in which keratinocytes are organized into basal, spinous, granular and cornified layers (Niessen et al., 2025). During development and homeostasis, basal keratinocytes generate suprabasal progeny that progressively differentiate as they move upward toward the surface, establishing the multilayered architecture of the tissue (Hsu and Fuchs, 2022). This process is accompanied by spatial polarization of cytoskeletal and junctional organization. Tissue cohesion relies on the coordinated assembly and remodeling of these junctional complexes. Adherens junctions couple adhesion to the actomyosin cytoskeleton, desmosomes anchor keratin intermediate filaments, and tight junctions in the granular layer form the permeability barrier (Niessen et al., 2025; Simpson et al., 2011; Sumigray and Lechler, 2015). These systems are functionally interdependent, as desmosome and tight junction assembly require prior adherens junction formation (Sumigray and Lechler, 2015). Crosstalk between actomyosin and keratin networks maintains the balance between contractility and mechanical resistance required for morphogenesis and barrier integrity (Javed et al., 2025; Rübsam et al., 2023). However, how cytoskeletal organization is coupled to junction remodeling during stratification remains unclear.

Unlike simple epithelia, stratified epithelia lack a canonical zonula adherens, with adherens junctions distributed along the entire cell–cell interface. In keratinocytes, E-cadherin-mediated adherens junctions form transient zipper-like contacts associated with radial actin fibers that mature into linear junctions linked to cortical actin bundles (Vaezi et al., 2002; Vasioukhin et al., 2000). During differentiation, junction-associated actin is extensively remodeled, including via desmoglein 1 (DSG1), a desmosomal cadherin and cortactin-Arp2/3-mediated polymerization, which reduces actomyosin contractility at E-cadherin complexes and facilitates the cell rearrangements required for delamination and stratification (Nekrasova et al., 2018). As cells enter suprabasal layers, they must re-establish junctions within a distinct mechanical environment. Protein kinase C iota type (PRKCI; also known as aPKCλ) signaling contributes to this transition by promoting reorganization of stress fibers into cortical actomyosin networks (Rübsam et al., 2023). Accordingly, suprabasal cells acquire a specialized actin architecture characterized by an apical F-actin meshwork, whereas keratin filaments dynamically adapt to changes in contractility to maintain tissue integrity (Rübsam et al., 2023). These features point to the need for mechanisms that fine-tune actomyosin tension during stratification.

Rho family GTPases are central regulators of cytoskeletal dynamics and junction assembly (Arnold et al., 2017). In keratinocytes, RAC1 and RHOA control cell–cell contact formation and associated actin remodeling (Braga et al., 1997; Vaezi et al., 2002), whereas CDC42 promotes junction maturation, and its loss impairs aPKC-dependent signaling and compromises skin barrier function in mice (Du et al., 2009; Wu et al., 2006; Zhang et al., 2019). Although these findings highlight the importance of Rho GTPase signaling in coordinating junctional organization and mechanical forces, how this activity is spatially and temporally regulated in the stratified epidermis remains unclear. To identify regulators of cytoskeletal dynamics and junction remodeling during epidermal morphogenesis, we previously performed an *in vivo* RNAi screen targeting Rho GTPase signaling components in embryonic epidermal progenitors (Laurin et al., 2019). Among the candidates identified was FYVE, RhoGEF and PH domain-containing 1 (FGD1), a CDC42 guanine nucleotide exchange factor (Olson et al., 1996; Zheng et al., 1996). FGD1-depleted cells were selectively lost from the mature epidermis, suggesting a role in tissue organization. Although few effectors of FGD1 have been defined, it interacts with cortactin and Abp1 (also known as AOC1) at the cortical actin cytoskeleton and localizes to the trans-Golgi network, where it recruits CDC42 (Egorov and Polishchuk, 2019; Hou et al., 2003). Mutations in FGD1 cause Aarskog–Scott syndrome (AAS), an X-linked developmental disorder associated with a combination of skeletal and genital abnormalities, with cutaneous features including altered skin elasticity and interdigital webbing (Kuo et al., 2022; Pasteris et al., 1994). The diversity of pathogenic variants identified in individuals with this condition suggests that variants differentially affect FGD1 activity (Zanetti Drumond et al., 2021). Although AAS is not considered a skin barrier disorder, these features raise the possibility that FGD1 contributes to epithelial organization.

Using an *in vitro* keratinocyte stratification model, we show that FGD1 promotes suprabasal junction maturation by organizing actin architecture and restraining actomyosin contractility. Loss of FGD1 disrupts the balance between the apical F-actin meshwork and radial actin bundles, increasing junction tortuosity and altering tissue mechanics. Several disease-associated FGD1 variants fail to restore normal junction organization, linking defective cytoskeletal–junction coupling to impaired epithelial integrity.

## RESULTS AND DISCUSSION

### FGD1 is required for cell–cell junction organization in differentiated keratinocytes

FGD1 was identified as a candidate regulator of epidermal morphogenesis in our *in vivo* RNAi screen (Laurin et al., 2019), and western blotting confirmed its expression in mouse embryonic epidermis (Fig. S1A). To investigate its function, we used hTERT-immortalized human foreskin keratinocytes (HFKs), which form adherens junctions and stratify upon CaCl_2_-induced differentiation (Fig. 1A) (Farwell et al., 2000). Reverse transcription real-time quantitative PCR (RT-qPCR) showed stable *FGD1* mRNA throughout differentiation (Fig. S1B), whereas western blotting revealed a transient increase in protein (Fig. 1B). Differentiation was confirmed by keratin type I cytoskeletal 10 (KRT10) induction, whereas keratin type II cytoskeletal 5 (KRT5) expression persisted, consistent with the presence of undifferentiated cells within the culture. Because endogenous FGD1 could not be detected by immunofluorescence, we localized 3×FLAG-FGD1 together with α-catenin as a junctional marker. FGD1 localized to the cytoplasm and cell cortex of both basal- and suprabasal-like cells (Fig. 1C).

**Fig. 1.**
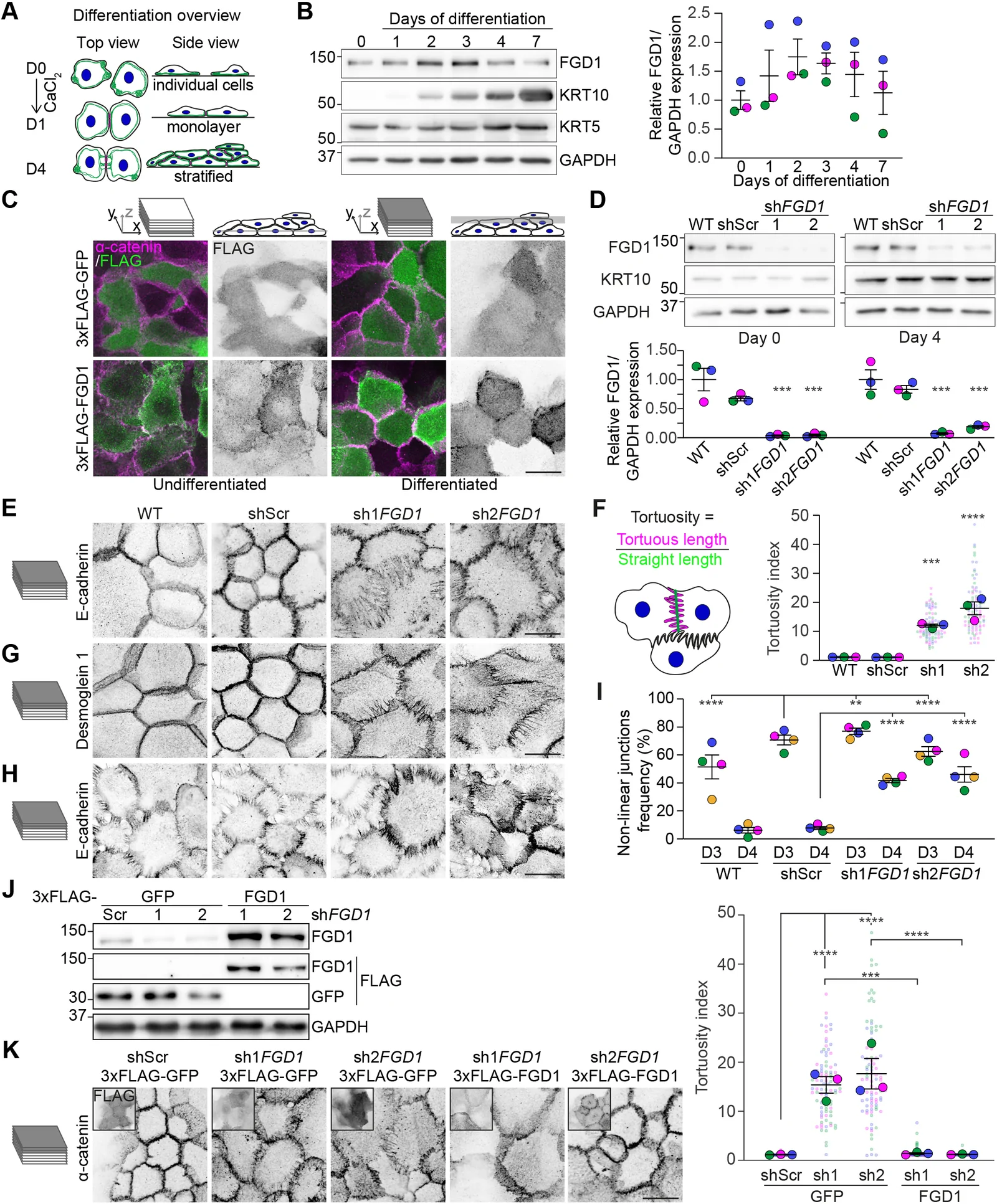
**FGD1 is required for cell–cell junction maturation in differentiated keratinocytes.** (A) Schematic of keratinocyte differentiation. (B) Western blot of FGD1, KRT10 and KRT5 during HFK differentiation; KRT5, basal-like cells; KRT10, differentiated cells; GAPDH, loading control. FGD1 levels are shown in the graph (GAPDH-normalized, relative to day 0; mean±s.e.m.; one-way ANOVA, *N*=3, *P*=0.5843; Dunnett's versus day 0, α=0.05). (C) Immunofluorescence of α-catenin and FLAG in HFKs expressing 3×FLAG–GFP or 3×FLAG–FGD1 at day 4 of differentiation: single optical section (basal, undifferentiated) and maximum-intensity projection of the apical *z*-stack (suprabasal, differentiated) (schematic). Images representative of three independent repeats. (D) Western blot of day 0 and day 4 HFKs expressing shScr, sh1*FGD1* or sh2*FGD1*; KRT10 confirms differentiation; GAPDH, loading control. FGD1 levels are shown in the graph below (GAPDH-normalized, relative to WT; mean±s.e.m.; one-way ANOVA, *N*=3; Dunnett's versus WT, α=0.05: day 0, *P*=0.0003; day 4, *P*=0.0002). (E) Immunofluorescence of E-cadherin at day 4. (F) Tortuosity index schematic and quantification at day 4 (mean±s.e.m.; nested one-way ANOVA, *N*=3, *n*=90 junctions/condition, Dunnett's versus shScr, α=0.05). (G) Immunofluorescence of desmoglein 1 at day 4. Images representative of three independent repeats. (H) Immunofluorescence of E-cadherin at day 3. (I) Non-linear junction frequency at days 3–4 [mean±s.e.m.; chi-square, *N*=4; day 3, *P*<0.0001 (WT *n*=1159, shScr=790, sh1*FGD1*=1059, sh2*FGD1*=1104), day 4, *P*<0.0001 (WT *n*=842, shScr=1000, sh1*FGD1*=866, sh2*FGD1*=913); Fisher's exact versus shScr per time point, Bonferroni α=0.01]. (J) Western blot of 3×FLAG-GFP or 3×FLAG-FGD1 at day 4; FGD1 detects endogenous and exogenous protein, FLAG detects exogenous constructs (FGD1 or GFP), GAPDH is loading control. Blots representative of three independent repeats. (K) Immunofluorescence of α-catenin and FLAG at day 4 (insets, FLAG). Graph on the right shows the tortuosity index (mean±s.e.m.; nested one-way ANOVA, *N*=3, *n*=90 junctions/condition, *P*<0.0001; Šídák's, α=0.05) versus shScr-3×FLAG–GFP; and 3×FLAG–FGD1 versus 3×FLAG–GFP. Scale bars: 20 μm. **P<0.01; ***P<0.001; ****P<0.0001. Exact *P*-values of multiple comparisons are available in Table S1.

To assess FGD1 function, HFKs were transduced with two independent *FGD1* shRNAs or a scrambled control (shScr). Western blot confirmed efficient depletion throughout differentiation (>90% and >80% for sh1*FGD1* and sh2*FGD1,* respectively) (Fig. 1D). FGD1 depletion did not affect proliferation or expression of differentiation markers *KRT5*, *KRT10*, loricrin and filaggrin (Fig. S1C,D). Control and FGD1-depleted cells formed comparable monolayers with linear E-cadherin junctions and cortical actin bundles at day 1 of differentiation (Fig. S1E,F). Likewise, basal keratinocytes remained morphologically similar at day 4 of differentiation (Fig. S1G). In contrast, defects emerged in suprabasal keratinocytes at this stage. Control cells formed linear continuous E-cadherin junctions, whereas FGD1-depleted cells displayed tortuous zipper-like junctions (Fig. 1E,F). DSG1 exhibited a similarly irregular distribution, consistent with membrane interdigitation (Fig. 1G). Zipper-like junctions were initially prevalent (∼80%) in both control and FGD1-depleted suprabasal cells at day 3 of differentiation, indicating that they represent a normal intermediate in junction maturation (Fig. 1H,I). By day 4, zipper-like junctions had largely resolved in wild-type (WT) and shScr cultures, decreasing by 88% and 89%, respectively. In contrast, they persisted in FGD1-depleted cells, declining by only 46% and 26% and remaining present in >40% of junctions in both knockdown conditions (Fig. 1I). These results indicate that FGD1 is dispensable for adherens junction formation but is required for their maturation into linear junctions in suprabasal keratinocytes. Consistent with this conclusion, re-expression of shRNA-resistant 3×FLAG–FGD1, but not 3×FLAG–GFP, restored junction linearity (Fig. 1J,K), demonstrating that the phenotype is caused by FGD1 depletion.

### FGD1 restrains actomyosin contractility in suprabasal keratinocytes

Tortuous junctions are associated with increased actomyosin contractility (Delsin et al., 2023; Javed et al., 2025; Rübsam et al., 2023; Sumi et al., 2018). We therefore tested whether elevated contractility underlies this phenotype. Treatment with the myosin II inhibitor blebbistatin restored linear E-cadherin junctions and reduced junction tortuosity in FGD1-depleted cells (Fig. 2A). Similarly, the ROCK inhibitor Y-27632 reduced junction tortuosity to control levels (Fig. 2B). Together, these findings suggest that FGD1 restrains ROCK-dependent actomyosin contractility during suprabasal junction maturation. Because junctional tension promotes α-catenin activation and vinculin recruitment (Javed et al., 2025; Yonemura et al., 2010), we examined α-catenin–vinculin organization. FGD1 depletion caused a modest reduction in vinculin levels, whereas α-catenin–vinculin colocalization showed a non-significant downward trend (Fig. 2C). Consistent with *in vivo* observations in the spinous layer (Rübsam et al., 2017), vinculin staining remained weak across all conditions.

**Fig. 2.**
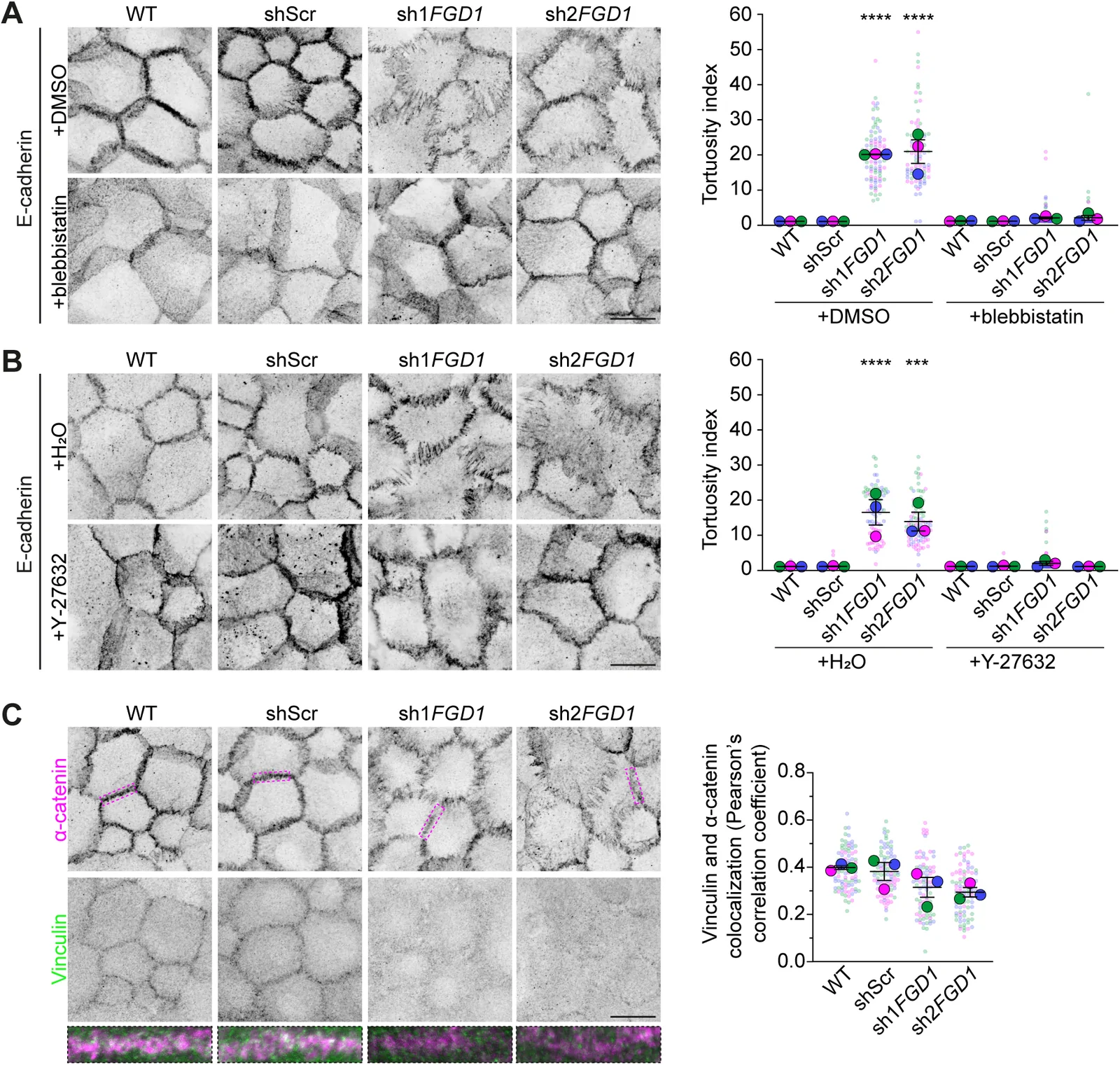
**FGD1 balances junctional tension and actomyosin contractility.** (A,B) E-cadherin immunofluorescence in HFKs at day 4 of differentiation treated with blebbistatin or vehicle (A) or Y-27632 or vehicle (B). Graphs to the right show tortuosity index for the blebbistatin experiment (mean±s.e.m.; nested one-way ANOVA, *N*=3, *n*=90 junctions/condition, *P*<0.0001; Šídák's versus corresponding shScr, α=0.05), and the tortuosity index for the Y-27632 experiment (same test versus corresponding shScr, *N*=3, *n*=90 junctions/condition, *P*<0.0001, α=0.05). (C) Immunofluorescence of α-catenin and vinculin at day 4 of differentiation. The graph to the right shows junctional α-catenin–vinculin colocalization (nested one-way ANOVA, *N*=3, *n*=90 junctions/condition, *P*=0.1087; Dunnett's versus shScr, α=0.05). Scale bars: 20 μm. Images are maximum-intensity projections of apical *z*-stacks. ***P<0.001; ****P<0.0001. Exact *P*-values of multiple comparisons are in Table S2.

### FGD1 organizes suprabasal actin architecture

To determine how FGD1 regulates junction architecture, we examined the suprabasal actin cytoskeleton. Whereas adherens junction maturation in monolayers involves cortical actin bundles (Efimova and Svitkina, 2018; Javed et al., 2025; Vasioukhin et al., 2000), suprabasal keratinocytes develop a distinct apical F-actin meshwork (Rübsam et al., 2017). By day 4 of differentiation, control suprabasal cells displayed this meshwork with short radial filaments extending toward linear E-cadherin junctions (Fig. 3A). FGD1 depletion disrupted this organization, producing elongated radial bundles that extended into membrane interdigitations and increased radial filament length by ∼3-fold (Fig. 3A). These bundles formed stellate-like cytoplasmic structures spanning adherens junctions, resembling the stress fiber-anchored, tension-associated ‘discontinuous’ or ‘focal’ adherens junctions described in endothelial cells (Huveneers et al., 2012; Millán et al., 2010). Although FGD1 depletion did not change total apical phosphorylated MLC2 (pMLC2; MLC2 is also known as MYL9) fluorescence intensity, it markedly altered its subcellular distribution, with pMLC2 becoming enriched along the elongated actin bundles (Fig. 3B), consistent with localized actomyosin contractility. Because FGD1 activates CDC42, we inhibited CDC42 with ML141. Although ML141 disrupted junction organization, it produced a more heterogeneous phenotype than FGD1 depletion, consistent with broader inhibition of CDC42 signaling. Whereas some cells developed tortuous junctions with radial actin bundles similar to those observed in FGD1-depleted cells, others displayed diffuse E-cadherin staining and lamellipodia-like protrusions (Fig. 3C). In contrast, Arp2/3 inhibition with CK-666 reproduced the FGD1 depletion phenotype, producing tortuous junctions associated with elongated actin bundles (Fig. 3D,E). Together, these findings support a model in which FGD1 promotes suprabasal junction maturation by regulating Arp2/3-dependent actin assembly. Given the established interaction between FGD1 and the Arp2/3 regulator cortactin (Hou et al., 2003; Kim et al., 2004), it might contribute to this pathway. However, we cannot exclude the possibility that FGD1 regulates Arp2/3 through localized CDC42 activation of N-WASP.

**Fig. 3.**
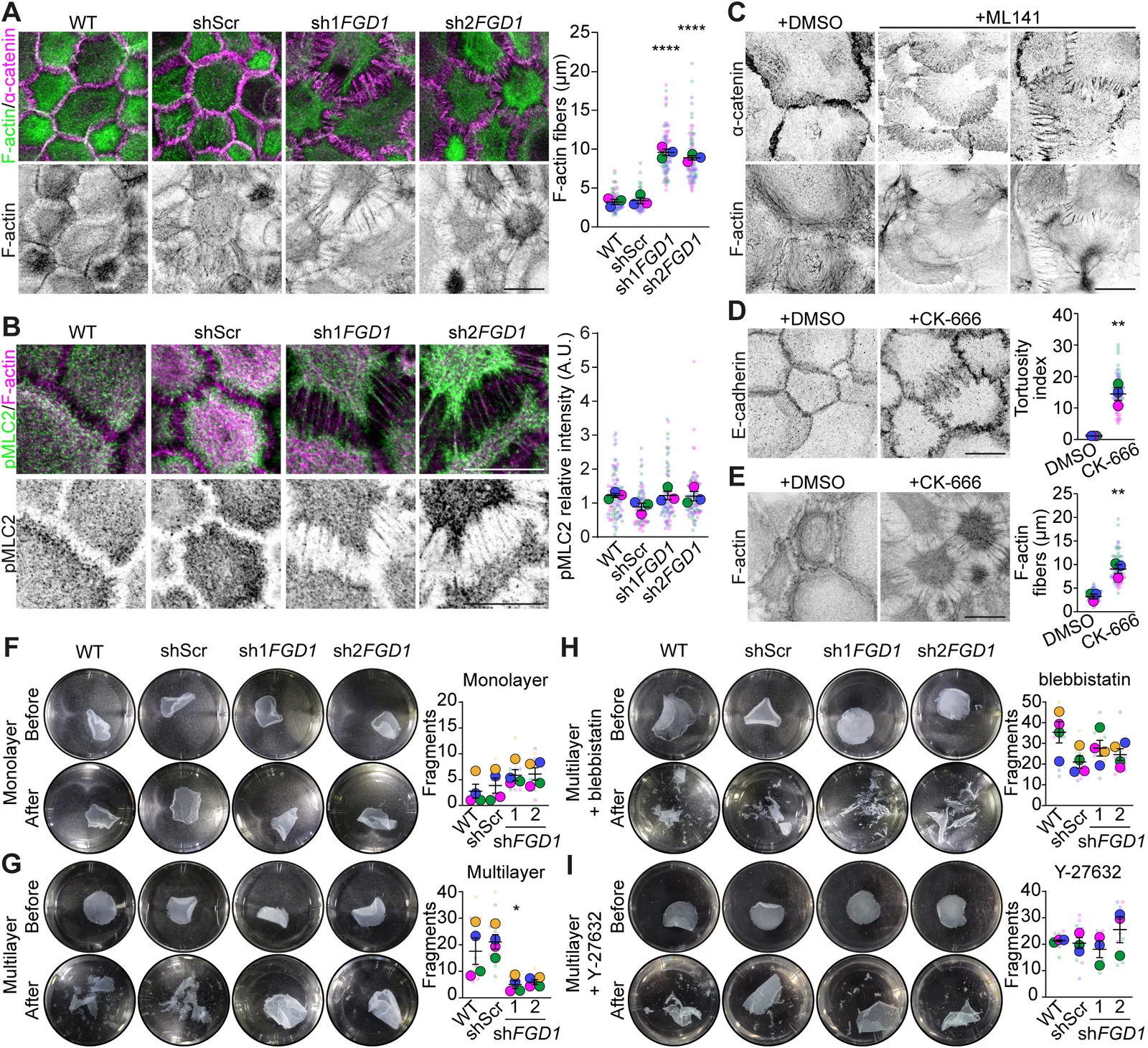
**FGD1 regulates suprabasal actin architecture and epithelial mechanics.** (A) Immunofluorescence of α-catenin and phalloidin-labeled F-actin at day 4 of differentiation. Graph shows radial F-actin length at junctions (mean±s.e.m.; nested one-way ANOVA, *N*=3, *n*=90 junctions/condition, *P*<0.0001; Dunnett's versus shScr, α=0.05). (B) Immunofluorescence of pMLC2 and phalloidin-labeled F-actin at day 4 of differentiation. Graph shows apical cortex quantification of pMLC2 (nested one-way ANOVA, *N*=3, *n*=90 cells/condition, *P*=0.1726; Dunnett's versus shScr, α=0.05). (C) Immunofluorescence of α-catenin and phalloidin-labeled F-actin at day 4 of differentiation following CDC42 inhibition (ML141, 20 μM, 4 h or vehicle). Images representative of three independent repeats. (D,E) E-cadherin (D) and phalloidin-labeled F-actin (E) immunofluorescence in HFKs at day 4 of differentiation treated with CK-666 or vehicle. Graphs show tortuosity index (D; mean±s.e.m.; two-tailed nested unpaired *t*-test, *N*=3, *n*=90 junctions/condition, *P*=0.0028); radial F-actin length (E; mean±s.e.m.; two-tailed nested *t*-test, *N*=3, *n*=90 junctions/condition, *P*=0.0057). (F,G) Dispase-based dissociation assay at day 1 (F) or 4 (G) of differentiation; top row, sheets before dissociation. Graphs show number of fragments (mean±s.e.m.; Kruskal–Wallis, *N*=4, three technical replicates each; Dunn's versus shScr: day 1, *P*=0.3177; day 4, *P*=0.0022). (H) Dissociation assay as in G with blebbistatin or vehicle pre-treatment. Graphs show number of fragments (mean±s.e.m.; Kruskal–Wallis, *N*=4, three technical replicates each, *P*=0.2030; Dunn's versus shScr). (I) Dissociation assay as in G with Y-27632 or vehicle pre-treatment; number of fragments (mean±s.e.m.; Kruskal–Wallis, *N*=3, three technical replicates each, *P*=0.7045; Dunn's versus shScr). Scale bars: 20 μm. Field of view (F–I)=9.6 cm^2^ (entire well). Images are maximum-intensity projections of apical *z*-stacks. A.U., arbitrary units. *P<0.05; **P<0.01; ****P<0.0001. Exact *P*-values of multiple comparisons are in Table S3.

### FGD1 regulates stratified tissue mechanical resistance

Keratin filaments adapt to changes in actomyosin organization, contributing to tissue mechanical resilience (Rübsam et al., 2023). We therefore tested whether the inability of FGD1-depleted cells to reorganize actomyosin contractility alters tissue mechanics using a dispase-based assay (Calautti et al., 1998; Schmidt et al., 2022), in which sheet fragmentation inversely reflects mechanical resistance. At day 1 of differentiation, control and FGD1-depleted monolayers generated a similar number of fragments following light mechanical stress (Fig. 3F), consistent with the absence of detectable defects in adherens junction morphology (Fig. S1E,F). In contrast, by day 4 of differentiation, FGD1-depleted stratified sheets exhibited increased mechanical resistance, with sh1*FGD1* yielding a significant 74% reduction in fragmentation and sh2*FGD1* showing a comparable but non-significant decrease (Fig. 3G). Because E-cadherin loss does not significantly affect resistance in this assay (Rübsam et al., 2023), this increase is unlikely to result from junctional changes alone but instead reflects cytoskeletal remodeling in FGD1-depleted cells. Consistent with this interpretation, inhibition of myosin II with blebbistatin or of ROCK with Y-27632 restored fragmentation to control levels (Fig. 3H,I). These findings identify FGD1 as a negative regulator of actomyosin-driven tissue mechanical resistance and link suprabasal cytoskeletal organization to the mechanical properties of stratified epithelial sheets. A similar mechanical adaptation occurs upon loss of the CDC42 effector aPKCλ in stratified keratinocytes (Rübsam et al., 2023). Because FGD1 can activate CDC42 and regulate Par6–Par3–aPKCζ signaling to control microtubule dynamics (Egorov and Polishchuk, 2019), it will be important to determine whether altered CDC42–aPKC signaling underlies the increased mechanical resilience of FGD1-depleted stratified tissues.

### Disease-associated FGD1 mutations impair junction maturation

Mutations in FGD1 cause AAS and are distributed across multiple functional domains (Li et al., 2024). To assess their effects in a disease-relevant loss-of-function context, we introduced substitutions that are seen in individuals with AAS into FGD1 and expressed doxycycline-inducible, shRNA-resistant 3×FLAG-tagged variants in FGD1-depleted keratinocytes: S205I (proline-rich domain), R443L, R522H and R610Q (CDC42 GEF domain), and C782R (FYVE membrane-targeting domain) (Fig. 4A,B). Most variants recapitulated the cortical-to-cytoplasmic distribution of FGD1^WT^, whereas GFP and C782R exhibited similarly reduced cortical enrichment, consistent with defective membrane targeting (Figs 1C and 4C,D) (Takahashi et al., 2024). Functionally, only S205I restored junction linearity, whereas the CDC42 GEF- and FYVE-domain mutants failed to rescue the phenotype (Fig. 4E). Although the CDC42 GEF-domain mutants localized similarly to FGD1^WT^, they remained functionally defective, indicating that their loss of function is not simply a consequence of impaired membrane localization. In contrast, the AAS-associated S205I variant retained WT-like activity, demonstrating that not all disease-associated mutations impair FGD1 function during junction maturation. Together, these findings support a role for FGD1 in adherens junction organization and suggest that AAS-associated variants differentially affect FGD1 functions, potentially contributing to the phenotypic variability observed in AAS.

**Fig. 4.**
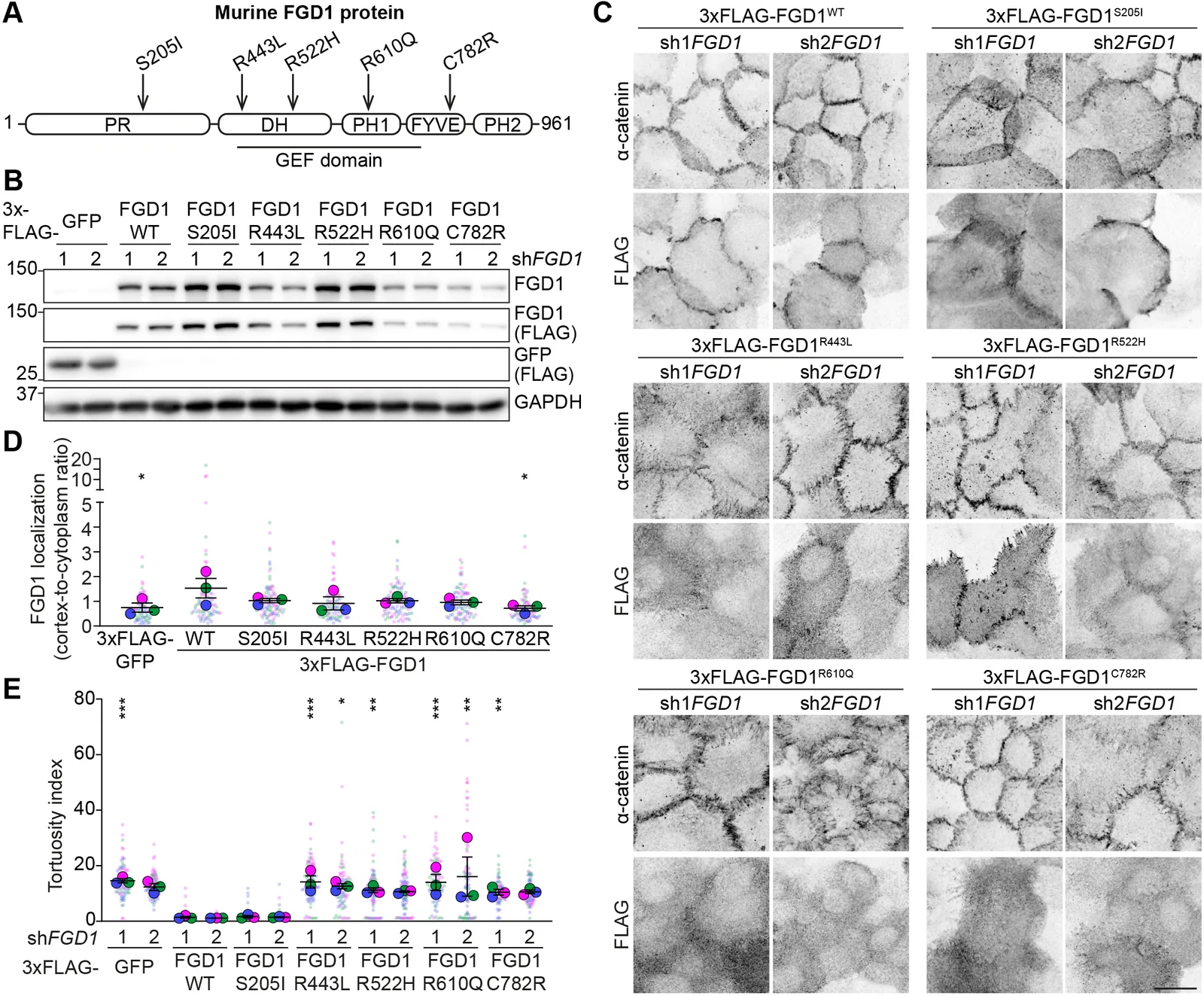
**Aarskog–Scott syndrome FGD1 variants fail to rescue junction defects.** (A) FGD1 domain organization and position of AAS-derived variants tested. (B) Western blot of doxycycline-inducible 3×FLAG–GFP, 3×FLAG–FGD1^WT^ and mutant constructs (S205I, R443L, R522H, R610Q, C782R) at day 4 of differentiation; FGD1 antibody detects endogenous and exogenous protein, FLAG detects exogenous constructs, GAPDH, loading control. Blots representative of three independent repeats. (C) Immunofluorescence of FLAG and α-catenin in HFKs expressing WT or mutant constructs at day 4 of differentiation. (D) FGD1 localization in HFKs expressing sh1*FGD1* and doxycycline-inducible 3×FLAG–GFP, 3×FLAG–FGD1^WT^ and mutant constructs (S205I, R443L, R522H, R610Q, C782R), shown as cortex-to-cytoplasm ratio (mean±s.e.m.; *P<0.1; outliers removed by Grubbs' method, α=0.0001; nested one-way ANOVA, *N*=3, *n*=90 cells/condition, *P*=0.1894; Dunnett's versus sh1*FGD1*-3xFLAG-FGD1^WT^, α=0.1). (E) Tortuosity index (mean±s.e.m.; *P<0.05; **P<0.01; ***P<0.001; nested one-way ANOVA per shRNA, *N*=3, *n*=90 junctions/condition; sh1*FGD1*, *P*<0.0001; sh2*FGD1*, *P*=0.0107; Dunnett's versus sh1*FGD1*-3xFLAG-FGD1^WT^, α=0.05 and versus sh2*FGD1*-3xFLAG-FGD1^WT^). Scale bars: 20 μm. Images are maximum-intensity projections of apical *z*-stacks. Exact *P*-values of multiple comparisons are in Table S4.

Our findings identify FGD1 as a key regulator of suprabasal junction remodeling that coordinates actin organization and contractility during epidermal stratification. By restraining actomyosin contractility, FGD1 promotes the transition from immature zipper-like contacts to linear adherens junctions. These findings support a model in which FGD1 balances Arp2/3-dependent actin networks and contractility to maintain junction architecture, epithelial cohesion and tissue mechanics in stratified epithelia. Future studies should define the downstream effectors of FGD1 and determine how it cooperates with other Rho GTPase regulators, including FGD family members, to control tissue morphogenesis and barrier function *in vivo*.

## MATERIALS AND METHODS

### Animal models

C57BL/6 mice (Charles River Laboratories) were maintained under specific pathogen-free conditions. Embryonic day (E)14.5–E15.5 skin lysates were prepared from pooled samples of both sexes. All animal procedures were approved by the Université Laval Animal Care and Use Committee and conducted in accordance with Canadian Council on Animal Care guidelines.

### Western blotting

Proteins were extracted from cultured cells or mouse tissues using RIPA buffer composed of 10 mM Tris-HCl pH 8.0, 1 mM EDTA, 0.5 mM EGTA, 1% Triton X-100, 0.5% sodium deoxycholate, 0.1% SDS and 140 mM NaCl, supplemented freshly with protease inhibitors (1× cOmplete EDTA-free protease inhibitor cocktail, Roche, 11836170001), 0.1 mM 4-(2-aminoethyl)benzenesulfonyl fluoride hydrochloride (AEBSF; Sigma-Aldrich, A8456) and 1 mM DTT. After 15 min on ice, cultured cells were collected by scraping, whereas mouse tissues were homogenized mechanically with a pestle. Cell lysates were sonicated six times for 10 s at 15% amplitude, whereas mouse tissues were sonicated three times for 5 s at 10% amplitude. Lysates were then clarified by centrifugation at 21,130 ***g*** for 10 min at 4°C. Proteins were resolved by SDS-PAGE on 8% polyacrylamide gels and transferred to 0.45 μm nitrocellulose membranes (Cytiva, 10600003). Membranes were incubated overnight with primary antibodies, washed 3×10 min in PBS-0.3% Tween-20, and incubated for 1 h with secondary antibodies. Following three washes of 10 min each, a chemiluminescent reaction was performed with Clarity Western ECL substrate (Bio-Rad, 1705060). Blots were imaged using an Amersham ImageQuant800 CCD imaging system (Cytiva, 29399481) following chemiluminescent detection (binning 1×1). Band intensities were quantified using the Gel Analysis tool in Fiji (ImageJ). FGD1 signals were normalized to GAPDH and expressed relative to the mean of the experimental control condition. Primary antibodies used were as follows: FGD1 (Sigma-Aldrich, HPA00091; 1:1000; RRID:AB_1078853), keratin 5 (Fuchs lab gift, rabbit; 1:10,000), FLAG M2 (Sigma-Aldrich, F1804; 1:1000; RRID:AB_262044) and GAPDH (Invitrogen, 39-8600; 1:10,000; RRID:AB_2533438). Secondary antibodies used were as follows: anti-mouse IgG HRP (Sigma-Aldrich, A9044; 1:15,000; RRID:AB_2772098) and anti-rabbit IgG HRP (Jackson ImmunoResearch, 111-035-144; 1:15,000; RRID:AB_2307391). Full uncropped images of western blots can be seen in Fig. S2.

### DNA constructs

shRNAs targeting human FGD1 (RefSeq NM_004463.3) or a non-targeting scrambled control were generated by annealing complementary oligonucleotides (listed below) and cloning them into the hygromycin-resistant pLKO.1 vector. shScramble primers: 5′-CCGGTGCGCGATAGCGCTAATAATTTTAGTGAAGCCACAGATGTAAAATTATTAGCGCTATCGCGCTTTTTG-3′ and 5′-AATTCAAAAAGCGCGATAGCGCTAATAATTTTACATCTGTGGCTTCACTAAAATTATTAGCGCTATCGCGCA-3′. sh1*FGD1* (CDS) primers: 5′-CCGGTGAGCGCTCCACCCAGTTTAAATAGTGAAGCCACAGATGTATTTAAACTGGGTGGAGCGCTCTTTTTG-3′ and 5′-AATTCAAAAAGAGCGCTCCACCCAGTTTAAATACATCTGTGGCTTGACTATTTAAACTGGGTGGAGCGCTCA-3′. sh2*FGD1* (3′UTR) primers: 5′-CCGGTGTTGCCAGTCCTACCTGAATTATAGTGAAGCCACAGATGTATAATTCAGGTAGGACTGGCTTCTTTTTG-3′ and 5′-AATTCAAAAAGAAGCCAGTCCTACCTGAATTATACATCTGTGGCTTCACTATAATTCAGGTAGGACTGGCAACA-3′. For rescue experiments, mouse FGD1 (RefSeq NM_001420098.1) was subcloned from mCherry–FGD1 (Addgene 129612) into pDONR221. An shRNA-resistant mouse FGD1 construct was generated by introducing silent mutations using the Q5 site-directed mutagenesis kit (New England Biolabs, E0554S). shRNA-resistant FGD1 and GFP coding sequences were then transferred into the doxycycline-inducible, puromycin-resistant pCW57.1-3xFLAG N-terminal vector using Gateway cloning with Gateway BP Clonase II (Thermo Fisher Scientific, 11789020) and Gateway LR Clonase II (Thermo Fisher, 11791020). pLKO.1, pCW57.1, pDONR221 and pENTRY-GFP plasmids were kindly provided by Amélie Fradet-Turcotte (Centre de recherche du CHU de Québec – Université Laval, Canada). FGD1 mutants were generated from the pCW57.1-3xFLAG-FGD1 rescue construct using the Q5 Site-Directed Mutagenesis Kit (New England Biolabs, E0554S). Equivalent positions of human AAS variants were identified in the mouse FGD1 sequence by alignment using the Standard Protein BLAST tool (NIH). The studied S205I, R443L, R522H, R610Q and C782R human mutations are, respectively, the equivalent of the S204I, R441L, R520H, R608Q and C780R mouse mutations used. Primers for FGD1 mutations are as follows: shRNA resistance (5′-ACAATTCAAGGTCATCATCCATGAAGTAC-3′ and 5′-GTGCTTCTTTCTGTCCATGTGTTGACCAG-3′); S205I (5′-AGCTGAGGCCATTACTAGTTCTGCAGC-3′ and 5′-CTGGGGACGAGTCCCTTG-3′); R443L (5′-GCTAGAGAAGCTGATGGAGGAATG-3′ and 5′-TCAGGCAGCAGGAAC-3′); R522H (5′-GCGTATCCCCCATTATGAACTTCTTCTCAAGG-3′ and 5′-TGCACGGGCTCCAGC-3′); R610Q (5′-CACTCAAGATCAGTACCTCATATTATTCAAC-3′ and 5′-GTCCCATTCTTTGCTG-3′); C782R (5′-CACTCAAGATCAGTACCTCATATTATTCAAC-3′ and 5′-GTCCCATTCTTTGCTG-3′).

### Keratinocyte culture, lentiviral production and infection

hTERT-immortalized human foreskin keratinocytes (HFK clone 22; Farwell et al., 2000) were provided by Aloysius J. Klingelhutz (University of Iowa, USA), tested for mycoplasma contamination, and maintained in keratinocyte serum-free medium (Keratinocyte-SFM; Gibco, 17005042) supplemented with 25 μg/ml bovine pituitary extract (Gibco, 13028-014), 0.16 ng/ml human recombinant epidermal growth factor (Gibco, 10450-013), 100 U/ml penicillin and 100 μg/ml streptomycin at 37°C in a humidified incubator with 5% CO_2_. Cells were maintained below 80% confluence to prevent spontaneous differentiation, and medium was changed every 3 days. Lentiviral particles were produced in HEK293T cells by co-transfection of psPAX2 (Addgene 12260) and pMD2.G (Addgene 12259) packaging plasmids together with transfer vectors encoding either shScramble, sh1*FGD1* and sh2*FGD1* cloned into the hygromycin-resistant pLKO.1 vector, or 3×FLAG–GFP and 3×FLAG–FGD1-shRNA-resistant cloned into the puromycin-resistant pCW57.1 vector. Viral supernatants were collected 48 h after transfection and filtered through 0.45 μm PES filters (Thermo Fisher Scientific, 13100107). For infection, 2×10^5^ HFKs per well were seeded in six-well plates 24 h before transduction. Viral supernatants were supplemented with 8 μg/ml polybrene (Sigma-Aldrich, 107689) and 10 mM HEPES (Gibco, 15630080), and up to 3 ml of viral supernatant was added per well. Cells were centrifuged at 200 ***g*** for 90 min at room temperature. After spinfection, viral supernatants were replaced with fresh Keratinocyte-SFM growth medium. The next day, infected cells were selected with 1 μg/ml hygromycin B (BioBasic, BS725) or 1 μg/ml puromycin (Sigma-Aldrich, P9620) for 3–5 days, with daily medium changes.

### Proliferation assay

HFKs were seeded at 10^3^ cells per well in 96-well plates in triplicate for each time point. A standard curve ranging from 10^3^–10^8^ cells was seeded in parallel to verify that the assay provided a sufficient dynamic range to detect proliferation changes. At the indicated times, Hoechst 33342 (Thermo Fisher Scientific, 62249) was added to a final concentration of 2 μg/ml and incubated for 30 min at 37°C. Fluorescent nuclei were imaged and quantified using a Cytation 5 Cell Imaging Multimode Reader (BioTek) and Gen5 software (Agilent BioTek).

### Keratinocyte differentiation assay

For RNA and protein analyses, 3×10^5^ HFKs were seeded per well in six-well plates. Differentiation was initiated 24 h later, at ∼80% confluence, by replacing the growth medium with Keratinocyte-SFM supplemented with 1.50 mM CaCl_2_ (final concentration 1.59 mM) and lacking growth factors. RNA and protein lysates were collected at day 1, 2, 3, 4 and 7 of differentiation. For immunofluorescence assays, 10^5^ HFKs were seeded onto 15 mm CultureWell chambered coverslips (Thermo Fisher Scientific, C24776), coated with poly-D-lysine (Thermo Fisher Scientific, A3890401). At the indicated time points, cells were washed with room-temperature PBS and fixed for 15 min with 4% paraformaldehyde (Electron Microscopy Sciences, EMS, 15710), followed by three washes in PBS before immunofluorescence staining. Expression of 3×FLAG constructs was induced using medium supplemented with 50 ng/ml doxycycline, with the medium changed daily. Myosin II, ROCK proteins, CDC42 and Arp2/3 were inhibited using 20 μM blebbistatin (1 h; Sigma-Aldrich, B0560), 10 μM Y-27632 (2 h; Cedarlane Labs, 10005583), 20 μM ML141 (4 h; Tocris Bioscience, 4266), or 100 μM CK-666 (2 h; Sigma-Aldrich, SML0006), respectively, prior to the end of differentiation.

### RNA isolation and RT-qPCR

RNA was isolated using the PureLink RNA Mini Kit (Invitrogen, 12-183-018A) according to the manufacturer's instructions, and 1 μg RNA was treated with DNase I (Thermo Fisher Scientific, EN0521) to remove gDNA and reverse transcribed using the High-Capacity cDNA Reverse Transcription Kit (Applied Biosystems, 4368814). Semi-quantitative PCR was performed using LightCycler 480 SYBR Green I Master Mix (Roche, 4707516001) with primers validated for their efficiency, as follows: *FGD1* (5′-AAAATGAACCCTTGGTGCT-3′ and 5′-GGCTGAAGTACCAGCTGAG-3′; 90.96%); *KRT5* (5′-ATCTCTGAGATGAACCGGATGATC-3′ and 5′-CAGATTGGCGCACTGTTTCTT-3′; 92.38%); *KRT10* (5′-TGGTTCAATGAAAAGAGCAAGGA-3′ and 5′-GGGATTGTTTCAAGGCCAGTT-3′; 90.20%); *FLG* (5′-ACTTCACTGAGTTTCTTCTGATGGTATT-3′ and 5′-TCCAGACTTGAGGGTCTTTTTCTG-3′; 111.20%); *LOR* (5′-AGGTTAAGACATGAAGGATTTGCAA-3′ and 5′-GGCACCGATGGGCTTAGAG-3′; 117.24%); *RPLP0* (5′-ACAACCCTGAAGTGCTTGATA-3′ and 5′-GACTCGTTTGTACCCGTTGA-3′; 91.50%); *GUSB* (5′-ATGGAAGAAGTGGTGCGTAG-3′ and 5′-CCTTGTCTGCTGCATAGTTAGA-3′; 83.64%), the latter two as housekeeping genes.

### Immunofluorescence, microscopy and image processing

Fixed cells were blocked for 1 h at room temperature in blocking buffer consisting of PBS, 2.5% normal donkey serum (Sigma-Aldrich, 566460), 1% bovine serum albumin (Wisent Bioproduct, 800-095-EG), and 0.1% Triton X-100. Primary antibodies were diluted in blocking buffer and incubated overnight at 4°C. Cells were then washed once with 0.3% Triton X-100 in PBS, followed by two washes in PBS. Secondary antibodies diluted in blocking buffer were applied for 1 h at room temperature with gentle agitation. After three washes in PBS, cells were incubated for 10 min with 200 ng/ml DAPI (Sigma-Aldrich, D9542) in PBS, followed by three additional PBS washes. Samples were mounted using ProLong Diamond Antifade Mounting Media (Invitrogen, P36961). When indicated, F-actin was co-stained using phalloidin–Rhodamine (Thermo Fisher Scientific, R415, 1:1000), phalloidin–Alexa Fluor 488 (Thermo Fisher Scientific, A-12379, 1:1000), or phalloidin-Alexa Fluor 647 (Thermo Fisher Scientific, A-22287, 1:1000) mixed with the secondary antibodies. Antibodies used are as follows: desmoglein 1 (R&D Systems, AF944; 1:200; RRID:AB_2277393), E-cadherin (BD Biosciences, 610181; 1:200; RRID:AB_397580), E-cadherin (R&D Systems, AF748; 1:100; RRID:AB_3555568), FLAG M2 (Sigma-Aldrich, F1804; 1:1000; RRID:AB_262044), α-catenin (Sigma-Aldrich, C2081; 1:500; RRID:AB_476830), vinculin (EMD Millipore, V9131; 1:500; RRID:AB_477629), pMLC2 (Cell Signaling, 3675; 1:100; RRID:AB_2250969). Secondary antibodies used were all purchased from Invitrogen, raised in donkey, and used at a 1:1000 dilution: anti-mouse IgG (H+L) Alexa Fluor 488 (R-37114; RRID:AB_2556542), anti-rabbit IgG (H+L) Alexa Fluor 488 (A-21206; RRID:AB_2535792), anti-goat IgG (H+L) Alexa Fluor 488 (A-11055;AB_2534102), anti-mouse IgG (H+L) Alexa Fluor 594 (A-21203; RRID:AB_141633), anti-rabbit IgG (H+L) Alexa Fluor 594 (A-21207; RRID:AB_1416637), anti-mouse IgG (H+L) Alexa Fluor 647 (A-31571; RRID:AB_162542), anti-rabbit IgG (H+L) Alexa Fluor 647 (A-31573; RRID:AB_2536183), anti-goat IgG (H+L) Alexa Fluor 647 (A-21447; RRID:AB_141844). Images were acquired using a Zeiss LSM 900 confocal microscope equipped with Plan-Apochromat 63×/1.40 Oil DIC M27 and Plan-Apochromat 40×/1.40 Oil DIC (UV) Vis-IR M27 objectives. Image processing was performed with Fiji (ImageJ; NIH) using only linear adjustments. For tortuosity analysis, the tortuous junction length and corresponding straight-line distance were measured using the segmented line tool in Fiji. The tortuosity index was calculated as the ratio between the tortuous length and the straight linear distance. Radial F-actin was quantified by measuring 10 fibers per junction, using the segmented line tool in Fiji. Fiber lengths were averaged by junction to obtain the mean F-actin radial length. Pearson correlation score of colocalization of vinculin and α-catenin was obtained using the BIOP JACoP plugin (https://imagej.net/plugins/jacop) on junctions selected with the Polygon selection tool in Fiji. Apical cortex intensity of pMLC2 was measured by delimiting the apical cortex of suprabasal cells on a sum-projection of a few *z*-sections with the freehand tool (Fiji). Integrated density of fluorescence was then normalized, for each cell, to the overall median for each experiment. For fluorescence intensity along cell borders, a straight line was drawn across the cell border, added to ROI manager and intensity was calculated with the MultiPlot tool. For quantification of FGD1 cortical enrichment, raw confocal images were background subtracted. A binary mask was generated from the membrane marker and expanded using successive dilatations to define cortical region. Whole-cell regions for FLAG-positive cells were manually delineated and the integrated fluorescence intensity of FGD1 was measured within the entire cell and within the cortical mask. Cortical enrichment was calculated as the ratio of integrated fluorescence within the cortical region to the integrated fluorescence outside the cortical region. All image processing and quantification steps were performed using custom Fiji macros (available at https://doi.org/10.5281/zenodo.22082847) to ensure identical analysis across all samples.

### Dispase-based keratinocyte dissociation assay

HFKs were seeded at 3×10^5^ cells per well in 12-well plates to obtain a uniform monolayer approaching 100% confluence prior to differentiation. Differentiation was induced 24 h after plating. At day 1 or 4, cells were incubated with Keratinocyte-SFM medium supplemented with 2.5 U/ml Dispase II (Sigma-Aldrich, D4693) at 37°C with 5% CO_2_ until sheets detached. The medium was then carefully removed, and 1 ml PBS was added per well. Mechanical stress was applied by repeated pipetting with a 1250 μl cut pipette tip. Five pipetting cycles were applied to day 1 monolayers, and 100 cycles were applied to day 4 stratified sheets. Fragments generated from dissociated sheets were counted manually. When indicated, cells were treated prior to dispase treatment with 20 μM blebbistatin (Sigma-Aldrich, B0560) for 1 h or 10 μM Y-27632 (Cayman Chemical, 10005583) for 2 h or their corresponding vehicle.

### Statistical analysis

Statistical analyses were all performed with Prism 8 (GraphPad Software). For the relative expression of mRNA in HFK cells, an unpaired two-tailed *t*-test between Day 0 and Day 7 was performed for *FGD1* mRNA transformed data of *N*=5 independent experiments (each with three technical replicates) with α=0.05. A one-way ANOVA between all cell lines was performed for each other mRNA (*KRT5*, *KRT10*, *LOR* and *FLG*) on day 7 transformed data of *N*=5 independent experiments (each with three technical replicates). Multiple comparisons were made with Dunnett's correction to assess the difference between each cell line compared to shScr and adjusted *P*-values are reported (with a starting α=0.05). For the expression of FGD1 protein, a one-way ANOVA was performed to assess differences in FGD1 expression between conditions for *N*=3 independent experiments. Multiple comparisons were made with Dunnett's correction to assess the difference between each condition compared to the appropriate experimental control (either HFK day 0 or HFK WT) and adjusted *P*-values are reported (with a starting α=0.05). For the frequency of non-linear cell–cell junctions, a Chi-square test was performed to assess the association between the junction type (linear or non-linear) and cell line in four independent experiments. Multiple 2×2 comparisons were made post hoc using two-sided Fisher's exact tests to assess the association of non-linear junctions in each condition compared to the appropriate experimental control, with a Bonferroni-adjusted α of 0.01. For the dissociation assays, Kruskal–Wallis tests were performed to assess differences between all conditions, with three (Y-27632) or four (24 h, 96 h, and blebbistatin) independent experiments. For proliferation assays, a Kruskal–Wallis test was performed to assess differences in proliferation rates at day 4 with four independent experiments and for quantification of fluorescence intensity along cell borders, analysis was performed on peak intensity values from 3 independent experiments. For all Kruskal–Wallis tests, multiple comparisons were made with Dunn's correction to assess the difference between each condition compared to the appropriate experimental control and adjusted *P*-values are reported (with a starting α=0.05). For the tortuosity index, the radial F-actin fiber length and apical cortex quantification of pMLC2, three independent experiments were analyzed with nested one-way ANOVA to assess differences between all conditions, except for CK-666 treatment, where a two-tailed nested *t*-test was performed on three independent experiments. Multiple comparisons were made with Dunnett's or Šídák's correction to assess differences between each condition and its appropriate control group, and adjusted *P*-values are reported (with starting α=0.05). For colocalization of vinculin and α-catenin, a nested one-way ANOVA was performed on Pearson's correlation coefficients obtained at the junctions from three independent experiments. For FGD1 cortex-to-cytoplasm ratio, data were cleaned of outliers using Grubbs' method (α=0.0001) and nested one-way ANOVA was performed with Dunnett's correction (with a starting α=0.1).

### AI use statement

Microsoft Copilot was used solely to assist with grammar and spelling corrections during manuscript preparation. The authors reviewed and verified all revisions and take full responsibility for the final content of the manuscript.

## Supplementary Material

10.1242/joces.264937_sup1 Supplementary information
