# Supplementary material for: FGD1 restrains actomyosin contractility to promote suprabasal junction maturation in stratified keratinocytes
Source: J Cell Sci. 2026 Sep 23;139(21):jcs264937. doi: 10.1242/jcs.264937 (PMC13680967; doi:10.1242/jcs.264937)
Supplement: Supplementary file 1 [file joces-139-264937-s1.pdf]

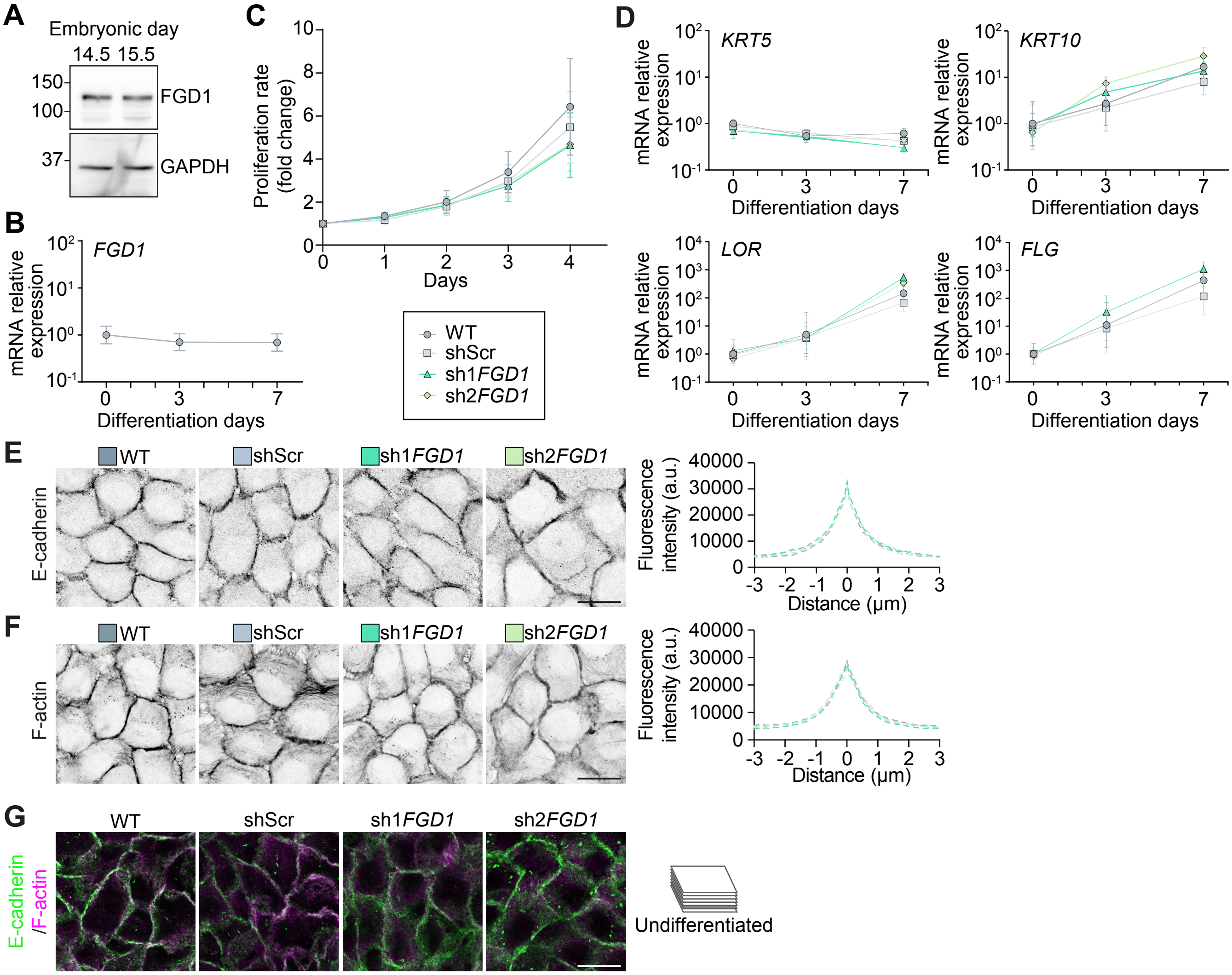

Fig. S1. FGD1 is dispensable for epidermal differentiation and monolayer organization. (A) Western blot of FGD1 in mouse skin lysates; GAPDH, loading control (representative of N=2 independent samples). (B) RT-qPCR of FGD1 expression during HFK differentiation (log_{10} scale; mean±SEM; unpaired two-tailed t-test, Day 0 vs Day 7, N=5, 3 technical replicates each, P=0.5577). (C) Cell proliferation (fold change in cell number) at Day 4 (mean±SEM; Kruskal-Wallis, N=4, 3 technical replicates each, P=0.8695; Dunn's vs shScr: WT, sh1*FGD1* and sh2*FGD1* P>0.9999). (D) RT-qPCR of *KRT5*, *KRT10*, *loricrin (LOR)* and *filaggrin (FLG)* during HFK differentiation (log_{10} scale; mean±SEM; one-way ANOVA at Day 7, N=5, 3 technical replicates each; Dunnett's vs shScr, α =0.05, adjusted P: *KRT5*, WT=0.6914, sh1*FGD1*=0.7387, sh2*FGD1*>0.9999; *KRT10*, WT=0.6302, sh1*FGD1*=0.8078, sh2*FGD1*=0.2347; *LOR*, WT=0.5768, sh1*FGD1*=0.0270 (*), sh2*FGD1*=0.0865; *FLG*, WT=0.6716, sh1*FGD1*=0.2874, sh2*FGD1*=0.6804). (E) Immunofluorescence of E-cadherin in HFK monolayers at Day 1 of differentiation; fluorescence intensity at cell borders (mean±SEM; Kruskal-Wallis, N=3, n=120 borders/condition, P=0.3059; Dunn's vs shScr: WT and sh2*FGD1* P>0.9999, sh1FGD1 P=0.2661). (F) Immunofluorescence of phalloidin-labeled F-actin in HFK monolayers at Day 1 of differentiation; fluorescence intensity at cell borders (mean±SEM; Kruskal-Wallis, N=3, n=120 borders/condition, P=0.1361; Dunn's vs shScr: WT and sh1*FGD1* P>0.9999, sh2*FGD1* P=0.5288); images representative of N=3 experiments. (G) Immunofluorescence of E-cadherin and phalloidin-labeled F-actin in basal layer of HFKs at Day 4 of differentiation. Scale bars: 20 µm. Immunofluorescence represents single optical section (basal, undifferentiated).

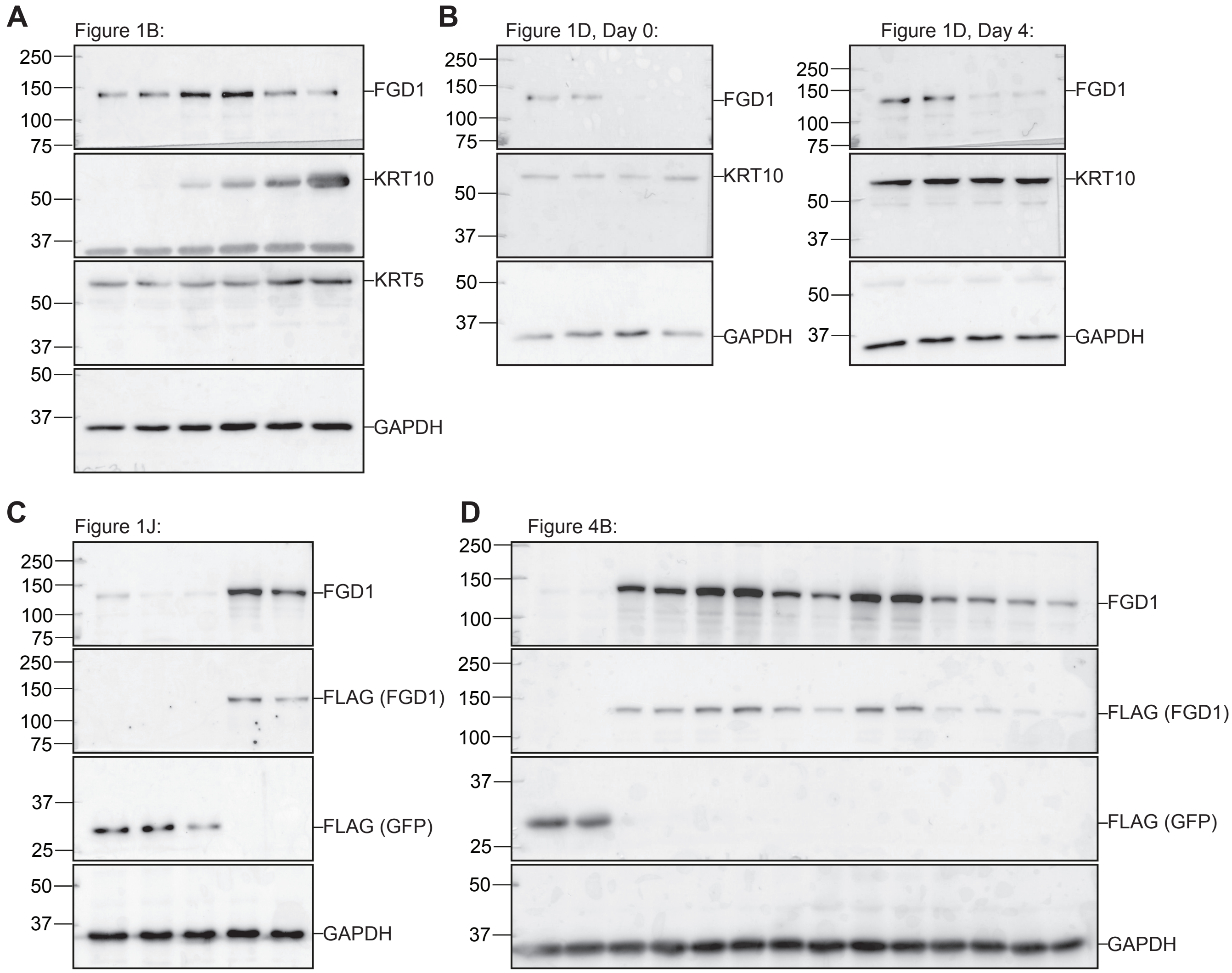

Fig. S2. Blot transparency. Uncropped Western blot of FGD1, FLAG, KRT10, KRT5, GAPDH, in HFK at various days of differentiation from Fig 1B (A), Fig 1D (B), Fig 1J (C) and Fig 4B (D).

Journal of Cell Science • Supplementary information

Table S1. Exact *P* values for multiple comparisons in Figure 1.

Figure 1B			
**Biological control**	**Sample**	**Statistical significance**	**Exact *P* value**
day 0	day 1	ns	0.8275
	day 2	ns	0.3914
	day 3	ns	0.5334
	day 4	ns	0.7975
	day 7	ns	0.9985

Figure 1D			
**Biological control**	**Sample**	**Statistical significance**	**Exact *P* value**
WT, day 0	shScr	ns	0.1160
	sh1*FGD1*	***	0.0004
	sh2*FGD1*	***	0.0004
WT, day 4	shScr	ns	0.4605
	sh1*FGD1*	***	0.0003
	sh2*FGD1*	***	0.0006

Figure 1F			
**Biological control**	**Sample**	**Statistical significance**	**Exact *P* value**
shScr	WT	ns	>0.9999
	sh1*FGD1*	***	0.0004
	sh2*FGD1*	****	<0.0001

Figure 1H			
**Biological control**	**Sample**	**Statistical significance**	**Exact *P* value**
shScr, day 3	WT	****	<0.0001
	sh1*FGD1*	**	0.0011
	sh2*FGD1*	****	<0.0001
shScr, day 4	WT	ns	0.1342
	sh1*FGD1*	****	<0.0001
	sh2*FGD1*	****	<0.0001

Figure 1K			
**Biological control**	**Sample**	**Statistical significance**	**Exact *P* value**
shScr-3xFLAG-GFP	sh1*FGD1*-3xFLAG-GFP	****	<0.0001
	sh2*FGD1*-3xFLAG-GFP	****	<0.0001
	sh1*FGD1*-3xFLAG-FGD1	ns	>0.9999
	sh2*FGD1*-3xFLAG-FGD1	ns	>0.9999
sh1*FGD1*-3xFLAG-GFP	sh1*FGD1*-3xFLAG-FGD1	****	0.0001
sh2*FGD1*-3xFLAG-GFP	sh2*FGD1*-3xFLAG-FGD1	****	<0.0001

Table S2. Exact *P* values for multiple comparisons in Figure 2.

**Figure 2A**			
**Biological control**	**Sample**	**Statistical significance**	**Exact *P* value**
shScr, DMSO	WT	ns	>0.9999
	sh1*FGD1*	****	<0.0001
	sh2*FGD1*	****	<0.0001
shScr, blebbistatin	WT	ns	>0.9999
	sh1*FGD1*	ns	0.9981
	sh2*FGD1*	ns	0.9978
WT DMSO	WT blebbistatin	ns	>0.9999
**Figure 2B**			
**Biological control**	**Sample**	**Statistical significance**	**Exact *P* value**
shScr, H_2O	WT	ns	>0.9999
	sh1*FGD1*	****	<0.0001
	sh2*FGD1*	****	0.0003
shScr, Y-27632	WT	ns	>0.9999
	sh1*FGD1*	ns	>0.9999
	sh2*FGD1*	ns	>0.9999
WT H_2O	WT Y-27632	ns	>0.9999
**Figure 2C**			
**Biological control**	**Sample**	**Statistical significance**	**Exact *P* value**
shScr	WT	ns	0.9608
	sh1*FGD1*	ns	0.3330
	sh2*FGD1*	ns	0.1702

Journal of Cell Science • Supplementary information

Table S3. Exact *P* values for multiple comparisons in Figure 3.

Biological control	Sample	Statistical significance	Exact *P* value
**Figure 3A**			
**Biological control**	**Sample**	**Statistical significance**	**Exact *P* value**
shScr	WT	ns	0.9766
	sh1*FGD1*	****	<0.0001
	sh2*FGD1*	****	<0.0001
**Figure 3B**			
**Biological control**	**Sample**	**Statistical significance**	**Exact *P* value**
shScr	WT	ns	0.1546
	sh1*FGD1*	ns	0.1548
	sh2*FGD1*	ns	0.1912
**Figure 3F**			
**Biological control**	**Sample**	**Statistical significance**	**Exact *P* value**
shScr	WT	ns	>0.9999
	sh1*FGD1*	ns	>0.9999
	sh2*FGD1*	ns	>0.9999
**Figure 3G**			
**Biological control**	**Sample**	**Statistical significance**	**Exact *P* value**
shScr	WT	ns	>0.9999
	sh1*FGD1*	*	0.0345
	sh2*FGD1*	ns	0.0637
**Figure 3H**			
**Biological control**	**Sample**	**Statistical significance**	**Exact *P* value**
shScr	WT	ns	0.1025
	sh1*FGD1*	ns	0.8944
	sh2*FGD1*	ns	>0.9999
**Figure 3I**			
**Biological control**	**Sample**	**Statistical significance**	**Exact *P* value**
shScr	WT	ns	>0.9999
	sh1*FGD1*	ns	>0.9999
	sh2*FGD1*	ns	>0.9999

Table S4. Exact *P* values for multiple comparisons in Figure 4.

Figure 4D			
**Biological control**	**Sample**	**Statistical significance**	**Exact *P* value**
sh1*FGD1*-3xFLAG-FGD1 WT	sh1*FGD1*-3xFLAG-GFP	*	0.0742
	sh1*FGD1*-3xFLAG-FGD1 S205I	ns	0.3708
	sh1*FGD1*-3xFLAG-FGD1 R443L	ns	0.2028
	sh1*FGD1*-3xFLAG-FGD1 R522H	ns	0.3570
	sh1*FGD1*-3xFLAG-FGD1 R610Q	ns	0.2560
	sh1*FGD1*-3xFLAG-FGD1 C782R	*	0.0615
**Figure 4E**			
**Biological control**	**Sample**	**Statistical significance**	**Exact *P* value**
sh1*FGD1*-3xFLAG-FGD1 WT	sh1*FGD1*-3xFLAG-GFP	***	0.0001
	sh1*FGD1*-3xFLAG-FGD1 S205I	ns	>0.9999
	sh1*FGD1*-3xFLAG-FGD1 R443L	***	0.0002
	sh1*FGD1*-3xFLAG-FGD1 R522H	**	0.0020
	sh1*FGD1*-3xFLAG-FGD1 R610Q	***	0.0002
	sh1*FGD1*-3xFLAG-FGD1 C782R	**	0.0038
**Biological control**	**Sample**	**Statistical significance**	**Exact *P* value**
sh2*FGD1*-3xFLAG-FGD1 WT	sh1*FGD1*-3xFLAG-GFP	ns	0.0527
	sh1*FGD1*-3xFLAG-FGD1 S205I	ns	>0.9999
	sh1*FGD1*-3xFLAG-FGD1 R443L	*	0.0431
	sh1*FGD1*-3xFLAG-FGD1 R522H	ns	0.1145
	sh1*FGD1*-3xFLAG-FGD1 R610Q	**	0.0085
	sh1*FGD1*-3xFLAG-FGD1 C782R	ns	0.1173

Journal of Cell Science • Supplementary information
